# Air pollutant prediction model based on transfer learning two-stage attention mechanism

**DOI:** 10.1038/s41598-024-57784-7

**Published:** 2024-03-28

**Authors:** Zhanfei Ma, Bisheng Wang, Wenli Luo, Jing Jiang, Dongxiang Liu, Hui Wei, HaoYe Luo

**Affiliations:** 1 School of Information Science and Technology, Baotou Teachers’ College, Baotou, 014010 Inner Mongolia China; 2 School of Information Engineering, Inner Mongolia University of Science and Technology, Baotou, 014010 Inner Mongolia China

**Keywords:** Climate sciences, Computer science

## Abstract

Atmospheric pollution significantly impacts the regional economy and human health, and its prediction has been increasingly emphasized. The performance of traditional prediction methods is limited due to the lack of historical data support in new atmospheric monitoring sites. Therefore, this paper proposes a two-stage attention mechanism model based on transfer learning (TL-AdaBiGRU). First, the first stage of the model utilizes a temporal distribution characterization algorithm to segment the air pollutant sequences into periods. It introduces a temporal attention mechanism to assign self-learning weights to the period segments in order to filter out essential period features. Then, in the second stage of the model, a multi-head external attention mechanism is introduced to mine the network's hidden layer key features. Finally, the adequate knowledge learned by the model at the source domain site is migrated to the new site to improve the prediction capability of the new site. The results show that (1) the model is modeled from the data distribution perspective, and the critical information within the sequence of periodic segments is mined in depth. (2) The model employs a unique two-stage attention mechanism to capture complex nonlinear relationships in air pollutant data. (3) Compared with the existing models, the mean absolute error (MAE), root mean square error (RMSE), and mean absolute percentage error (MAPE) of the model decreased by 14%, 13%, and 4%, respectively, and the prediction accuracy was greatly improved.

## Introduction

With the rapid growth of China's economy and the rapid development of industrialization, the pressure on the environment continues to increase, and severe air pollution has brought many inconveniences to people's lives; the relevant departments have begun to monitor the concentration of pollutants in the atmosphere. PM_2.5_, PM_10_, NO_2_, SO_2_, O_3_, and so on, can be suspended in the atmosphere due to their tiny and lightweight properties. These tiny particles can enter the body through the respiratory tract to reach the depths of the lungs, causing irreversible damage to the respiratory system. Studies have shown that long-term exposure to high concentrations of PM_2.5_ PM_10_ not only increases the risk of respiratory diseases but also cardiovascular will produce adverse damage; SO_2_ particles will cause great harm to the environment, deposition to the soil and water contamination of soil and water, which affects crops and vegetation. This not only affects the growth of crops and vegetation but also disrupts the balance of the ecosystem; prolonged exposure to high concentrations of NO_2_ and O_3_ can lead to symptoms such as coughing, dizziness, and reduced concentration. Therefore, predicting the concentration of air pollutants has an important guiding role for the government in controlling air pollution and formulating relevant environmental protection policies. It also has an important significance for improving people's quality of life.

Currently, there are two main methods for predicting atmospheric pollutant concentration: physicochemical and data-driven methods. Physicochemical methods predict changes in air pollutants at different scales and regions by modeling and analyzing the physicochemical reactions of air pollutants through physicochemical principles. They mainly include the Nested Air Quality Prediction Modeling System (NAQPMS), WRF_Chem model, and the Community Multiscale Air Quality (CMAQ) model^1,2^. Although these models can achieve a high prediction accuracy, they often require complex model configurations and parameter adjustments, and different numerical prediction models are required for different locations, resulting in low generalizability of the models.

With the gradual establishment of monitoring tools such as meteorological observation stations, atmospheric quality monitoring stations, and meteorological satellites, the atmospheric pollutant concentration data and meteorological data collected by the equipment provide data support for atmospheric quality prediction research. Data-driven methods are increasingly being applied to predict the concentration of atmospheric pollutants. In early statistical modeling, commonly used models include the autoregressive moving average model (ARMA), autoregressive Integrated moving average model (ARIMA), and multivariate linear regression model (MLR)^3^. Due to the influence of various factors on the concentration of atmospheric pollutants, they exhibit instability and nonlinearity. The above statistical modeling methods are not accurate in processing nonlinear sequence data, which in turn affects the prediction accuracy. In recent years, with the development of machine learning, methods such as multilayer perceptron (MLP)^4^, support vector machines (SVM)^5,6^, and random forest (RF)^7–9^ have been used for predicting atmospheric pollutants. Although traditional machine learning methods have achieved good results in predicting air pollution, the concentration of air pollutants not only has the characteristics of mutual conversion and cancellation but is also easily affected by meteorological factors, so it cannot effectively capture the time series characteristic information of air pollutant concentration. In order to extract internal feature correlation information from historical data, many scholars use neural networks to construct atmospheric pollutant prediction models, such as recurrent neural network (RNN)^10,11^, long short term memory (LSTM)^12–14^, weighted long short-term memory neural network (WLSTME)^15^, bi-directional long short-term memory neural network (BiLSTM)^16^, and gated recurrent unit (GRU)^17^. Some studies have combined the above networks to explore the long-term dependencies of data. For example, Huang et al.^18^ combined convolutional neural network (CNN) and long short-term memory network (LSTM) for PM_2.5_ concentration prediction. Du et al.^19^ established a combination model of CNN and BiLSTM for multivariate atmospheric quality prediction. Zhang et al.^20^ proposed a hybrid model based on residual network (ResNet) and convolutional long short-term memory network (ConvLSTM) to predict PM_2.5_ concentration in cities for a period of time in the future. Furthermore, a prediction model is constructed by combining coupled swarm intelligence algorithms with neural networks^21,22^, which have the characteristics of fewer dependent variables and higher prediction accuracy. The above deep learning-based prediction methods require sufficient training data. Otherwise, the trained neural network has poor robustness, low accuracy, and weak generalization ability. Leveraging its advantages for newly built atmospheric monitoring stations with limited historical data is difficult.

Some studies have noted that data interpolation methods to predict pollutants can alleviate the problems caused by data imbalance. For example, by interpolation, Chae et al.^23^ transformed non-uniform data from different monitoring locations into uniform spatial data. They combined it with the CNN model to construct an ICNN model for air quality prediction, which showed high prediction accuracy for PM_10_ and PM_2.5_. Samal et al.^24^ proposed a Multi-directional Temporal Convolutional Neural Network (MTCAN) model, the main idea of which is to use the correlation between pollutants and meteorological factors to fill in the missing values of PM_2.5_ and then combine it with the null convolutional features of the TCN model for prediction, and the results showed a significant improvement in the prediction accuracy of the proposed model. Ding et al.^25^ proposed a geographic long- and short-term memory neural network (Geo-LSTM) based on interpolation of air pollutant spatial distributions, which was compared with the traditional spatial interpolation methods and the machine learning-based interpolation methods; the proposed model not only can learn nonlinearly from the long-term dependence of time series but also takes into account the spatiotemporal mechanism of air pollutants. Recently, transfer learning (TL) has been widely used in computer vision^26^, text classification^27^, activity recognition^28^, multilingual speech technology^29^, and other fields because it can transfer learned knowledge to target fields to solve problems with a small amount of labeled sample data. In view of its unique performance in solving the small sample learning problem, some research attempts to apply TL to the prediction of atmospheric pollutant concentration series. For example, Ma et al.^30^ proposed a transfer learning-based stacked bidirectional long short term memory network (TLS-BLSTM) for predicting atmospheric quality at a new station lacking data, which transfers knowledge learned from an existing atmospheric quality station to the new station to improve forecasting capability. Using meteorological and pollutant concentration data as model inputs, Yuan et al.^31^ proposed a new model coupling long short-term memory neural network with transfer learning (TL-LSTM) to improve the accuracy and generalization ability of model prediction. Aiming at the problem of an existing single method for processing missing data, Ma et al.^32^ proposed an iterative estimation based on transferred long short-term memory-based iterative estimation (TLSTM-IE) for estimating consecutive missing values with large missing rates. However, the relationship between air pollutant variables is complex characterized by strong periodicity, continuity, and non-stationary. The above methods will have the problem of insufficient information mining of original data. Scholars have noticed that the prediction performance can be improved by improving the input variables, such as discrete wavelet transform (DWT), fourier transformed partial modulus Division (DFM), empirical mode decomposition (EMD), ensemble empirical mode decomposition (EEMD)^33^, complementary ensemble empirical mode decomposition (CEEMD)^34^, and so on, but these methods do not consider the impact of data distribution changes on the prediction results, which means that these methods may encounter the problem of model drift when facing unknown data, This will lead to insufficient training, which will affect the prediction accuracy.

To this end, this paper proposes an atmospheric pollutant prediction model (TL-AdaBiGRU) based on a two-stage attention mechanism of transfer learning. The prediction method is modeled from the perspective of air pollutant data distribution, using temporal distribution characterization to segment the air pollutant sequences periodically to capture the sequence period information fully and embedding a temporal attention mechanism layer and a multi-head external attention mechanism layer based on bidirectional gated recurrent neural network (BiGRU) to excavate long-term dependencies of time series deeply. Moreover, the BiGRU model incorporating the two-stage attention mechanism is combined with transfer learning for monitoring station prediction with limited historical data. The model proposed in this paper can effectively alleviate the problems of poor model generalization and poor prediction accuracy caused by data periodicity, non-stationarity, and insufficient data volume. The main contributions of this paper are as follows:

1. In this paper, the characteristics of air pollutant concentration data with strong periodicity, continuity, and non-stationarity are taken into account, and the TDC algorithm is utilized to segment the sequence and learn the characteristics between the periods.
2. In order to better mine the potential information of the input data and capture the complex features of the data, the temporal attention mechanism and multiple external attention mechanisms are embedded in the temporal distribution matching layer. Through the temporal attention mechanism layer, the importance of different periods is determined, and the corresponding weights are assigned to obtain a better model input. In order to dig deeper into the critical information in the hidden layer of BiGRU and extract the temporal characteristics between different units, the temporal dependence between units is captured by embedding a multi-head external attention mechanism layer after the BiGRU layer, which assigns different attention to the important information in the hidden layer, and then learns the critical information inside the model.
3. The BiGRU model incorporating a two-stage attention mechanism is combined with transfer learning, and the source domain data determined by the Multiple Kernel Maximum Mean Discrepancy (MK-MMD) is used to pre-train the model to determine the optimal network parameters. In the transfer phase, the target domain data is used to fine-tuning the pre-training model to improve the generalization ability further. Through comparative analysis of prediction performance on sites lacking historical data, the TL-AdaBiGRU model is superior to Transformer, AdaBiGRU, BiGRU, GRU, and LightGBM models in prediction effect.

## Air pollutants prediction approach

### Air pollutants prediction framework

**Figure 1:**
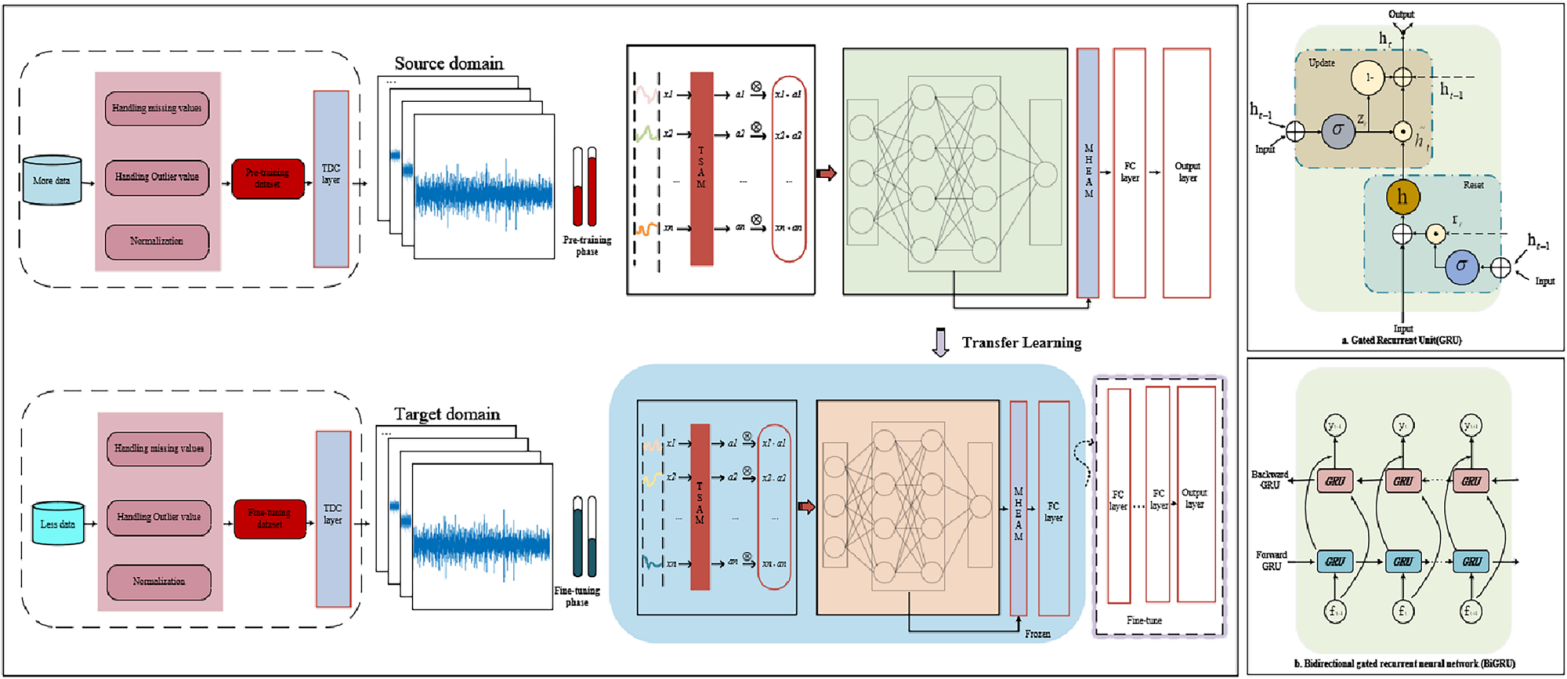
Air pollutants prediction framework. (**a**) is GRU structure, (**b**) is BiGRU structure.

The air pollutant concentration prediction framework proposed in this paper is shown in Fig. 1 below. It can be divided into a pre-training stage and a transfer stage. For the pre-training stage, firstly, the pollutant concentration data and meteorological data are detected anomalously, and the detected anomalies are marked as missing values and the linear interpolation algorithm is used to fill in the missing data, after which the data are normalized. Secondly, the preprocessed data are fed into the temporal distribution characterization layer (TDC). The design of the TDC is inspired by the principle of maximum entropy, which divides the time series into ten parts uniformly and uses a greedy strategy to divide the length $n_{j}$ of each cycle, thus dividing the data into $K$ periods with large distribution gaps. This design aims to reduce the effect of data periodicity and helps the model better learn each time period's internal information. Next, the first-stage attention mechanism-temporal attention mechanism is used to assign weights a to each temporal data $x_{i}$ according to the importance of the temporal data in order to pay full attention to the feature information in the time-series data. Finally, the product of each temporal data $x_{i}$ and the attention $\alpha$, $f_{i}$, is used as the input to the BiGRU network. The hidden layer of BiGRU can efficiently capture the sequence data's long-term dependencies and effectively fuse forward and backward information to generate more comprehensive and accurate feature representations. A second-stage attention mechanism, the Multihead External Attention Mechanism, is embedded behind the hidden layer of BiGRU to dig deeper into the key features of the network's hidden layer. The composition of the multi-head external attention mechanism consists of two independent memory units,$M_{K}$ and $M_{v}$, which are used as keys and values, respectively. They can learn additional data features and prior knowledge to assist the model in feature selection and weighting, quickly filtering out the key features among numerous inputs. Finally, the source domain pre-training is completed using the fully connected layer. In the transfer stage, the parameters from the pre-training phase are used as the basis for the transfer learning using the fine-tuning strategy. First, we froze the last four layers of the AdaBiGRU model and performed a certain number of Epoch training to verify the fitting effect of the source domain. Then, we unfroze the frozen layers and designed a new fully connected layer spliced with the unfrozen AdaBiGRU to obtain the new AdaBiGRU model. The new AdaBiGRU model contains the pre-trained AdaBiGRU layer of the source domain and the thawed AdaBiGRU layer (without weight update). Finally, we fine-tuned the AdaBiGRU model using the preprocessed target site data to optimize the remaining parameters. We applied the optimal TL-AdaBiGRU model to predict air pollutant concentrations at the target site and output the final prediction results.

### Two-stage attention mechanisms neural networks

We propose AdaBiGRU, consisting mainly of a temporal distribution characterization module (TDC) and a temporal distribution matching module (TDM). The role of the TDC module is to quantify the successive data distributions in a sequence and classify them into sequences with the least similar ${\text{K}}$ segment distributions. The role of the TDM module is to construct a model with temporal invariance for the above K-segment sequences. The details are given below.

### Temporal distribution characterization

**Figure 2:**
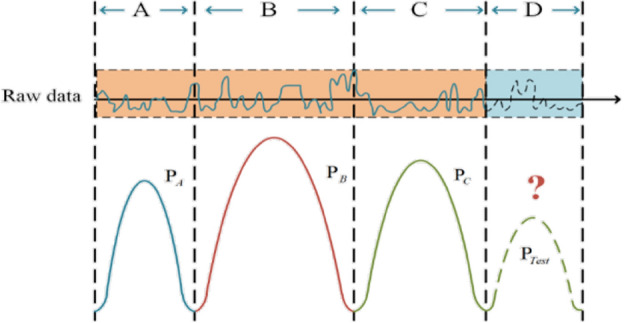
Temporal covariate shift.

Atmospheric pollutant concentration data are typical time series data with periodicity and non-stationarity, and the data distribution changes dynamically with time. This paper defines the problem above as Temporal Covariate Shift (TCS). TCS means that there are ${n}$ marked parts in a period of time ${\text{D}}$. If we can divide it into ${\text{K}}$ period segments, that is, ${D = }\left\{ {{D}_{{1}} } \right.{,D}_{{2}} {,}...{,}\left. {{D}_{{K}} } \right\}$, where $D_{K} = \left\{ {x_{i} ,y_{i} } \right\}_{{i = n_{K} + 1}}^{{n_{K + 1} }} ,n_{1} = 0,n_{K + 1} = n$. It is referred to the case that all the segments in the same period follow the same data distribution $P_{{D_{i} }} \left( {x,y} \right)$,while for different time periods $1 \le {\text{i}} \ne j \le K,P_{{D_{i} }} (x) \ne P_{Dj} (x)$ and $P_{{D_{i} }} \left( {{\text{y}}|x} \right) = P_{Dj} \left( {{\text{y}}|x} \right)$. As shown in Fig. 2 below, the data have different distributions in intervals A, B, C and D, that is,${\text{P}}_{A} \left( x \right) \ne {\text{P}}_{B} \left( x \right) \ne {\text{P}}_{C} \left( x \right) \ne {\text{P}}_{Test} \left( x \right)$. Especially during our training process, the distribution of the test data and the training data are also different, so how to solve the differences between the data distributions while capturing the common knowledge of the time series data between different periods to make the prediction model generalize more is the primary problem.

One approach of existing studies for the above scenario is to assume that all-time series segments follow the same data distribution, but this is clearly inappropriate in air pollutant prediction. Another approach is to use some adaptive algorithms to reduce the distributional differences between the data and thus learn the invariant knowledge of the data domain, such as Domain Adaptation (DA)^35^ and Domain Generalization (DG)^36,37^, which in turn are differentiated in that the former aims at reducing the distributional differences between the training data and the test data by learning a domain-invariant representation, and the latter hopes to learn a domain-invariant model over multiple source domains to learn a domain-invariant model which generalizes well to the target domain. Unfortunately, atmospheric pollutants are not only time-varying but also have a strong sequence structure, making it difficult for DA and DG methods to address the data distribution differences effectively.

In order to better represent the distribution information in the time series, this paper proposes a temporal distribution characterization (TDC) algorithm, which is described in detail in Section TDC. According to the principle of maximum entropy, the training data is partitioned into K time periods with large distribution intervals to train the model; when the prediction model can have good generalization between periods with significant differences in the data distribution, then the performance must also be better for periods with more minor differences in the distribution. TDC achieves the time series partitioning by solving an optimization problem, which can be formulated as follows:

1 $$ \begin{gathered} \mathop {max}\limits_{{0 < K < K_{0} }} \mathop {max}\limits_{{n_{1} ,...,n_{k} }} \frac{1}{K}\sum\limits_{{1 \le {\text{i}} \ne j \le K}} {d\left( {D_{i} ,D_{j} } \right)} \hfill \\ s.t.\quad \forall i,\Delta_{1} < \left| {D_{i} } \right| < \Delta_{2} ;\sum\limits_{i} {\left| {D_{i} } \right|} = n \hfill \\ \end{gathered} $$

where $${\Delta }_{{1}}$$$${,\Delta }_{{2}}$$ and $${K}_{{0}}$$ are pre-set parameters to avoid meaningless solutions.$${\text{d}}$$ selects CORAL as the similarity measure function, and the covariance distance of the distribution samples represented by CORAL is shown in Eq. (2).

2 $$ {d}_{{{coral}}} \left( {{h}_{{S}} {,h}_{{t}} } \right){ = }\frac{{1}}{{{4q}^{{2}} }}\left\| {{C}_{{S}} - {C}_{{t}} } \right\|_{{F}}^{{2}} $$

where ${q}$ is the dimension of the features and ${C}_{{S}} {,C}_{{t}}$ is the covariance matrix of the distribution.

### Temporal distribution matching

After the TDC module, which obtains the least similar sequences of ${\text{K}}$ segments, the TDM module assigns different temporal self-attention to the period sequences according to the importance of the period. In particular, in order to learn the temporal distribution properties and sequence correlations, AdaBiGRU adaptively matches the distributions among BiGRU units for each period using a multi-head external attention mechanism while capturing the temporal dependencies. The details are as follows.

### Temporal self-attention mechanism

In deep learning, the self-attention mechanism^38^ is a vital model structure used to improve the model's attention to and processing of input data. The self-attention mechanism allows the model to selectively focus on the essential parts and ignore the unimportant parts when processing the input data, thus improving the performance and effectiveness of the model. In this paper, we calculate the degree of correlation between each location of the input data and other locations through the temporal self-attention mechanism layer to get the weight of each location. By calculating the weights, the model can focus more on this task-relevant information and improve its processing power.

**Figure 3:**
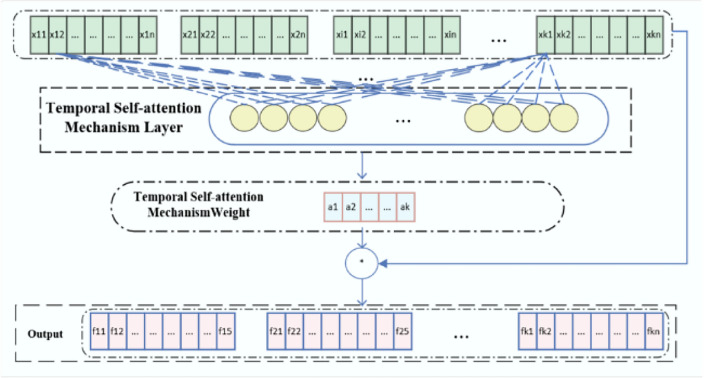
The temporal self-attention mechanism structure.

According to Eq. 1, a plurality of period segment data ${Z = }\left\{ {{z}\left( {t} \right)\left| {{t = d,d + 1,}...{,K}} \right.} \right\}$ is used as input to the TSAM layer. The data for each period segment can be represented as: ${z}\left( {t} \right){ = }\left[ {{x}_{{\left( {{t,1}} \right)}} {,x}_{{\left( {{t,2}} \right)}} {,}...{,x}_{{\left( {{t,d}} \right)}} } \right]{,x}_{{\left( {{t,1}} \right)}} \in {R}^{{m}} {,}\left( {{1,2,}...{,d}} \right){,d}$ is the length of each period. As shown in Fig. 3. Periodic data is passed through the TSAM layer to obtain a mapping relationship between time instances, as shown in Eqs. (3) and (4):

3 $$ {\gamma }_{{i}} { = \sigma }\left( {{W}_{{i}}^{{T}} {x}_{{i}} { + b}_{{i}} } \right){ = }\frac{{1}}{{{1 + e}^{{ - \left( {{W}_{{i}}^{{T}} {x}_{{i}} { + b}_{{i}} } \right)}} }} $$

4 $$ {\alpha }_{{i}} { = softmax}\left( {{\gamma }_{{i}} } \right){ = }\frac{{{e}^{{{\gamma }_{{i}} }} }}{{\sum\nolimits_{{{j = 1}}}^{{d}} {{e}^{{{\gamma }_{{j}} }} } }} $$

${x}_{{i}}$ denotes the ${i}$ th temporal data,${W}_{{i}}$ and ${b}_{{i}}$ denote the preset weights and biases corresponding to the ${i}$ th temporal data,${T}$ is the device operation,${\sigma }$ is the ${sigmoid}$ activation function, and ${a}_{{i}}$ denotes the temporal attention weight corresponding to the ${i}$ th temporal data.

Finally, the temporal attention weight ${a}_{{i}}$ corresponding to each temporal data is multiplied with the corresponding sample data ${x}_{{i}}$ to obtain the output ${f}_{{i}}$ of each period sample in the temporal self-attention mechanism layer, and the output ${F}$ of the whole temporal self-attention mechanism layer is used as the input of the subsequent BiGRU. As shown in Eq. (5).

5 $$ {F = }\left[ {{f}_{{1}} {,f}_{{2}} {,}...{,f}_{{d}} } \right]{ = }\left[ {{a}_{{1}} {x}_{{1}} {,a}_{{2}} {x}_{{2}} {,}...{,a}_{{d}} {x}_{{d}} } \right] $$

### Bidirectional gated recurrent neural network

Gated Recurrent Unit (GRU) is a Recurrent Neural Network (RNN) variant for processing sequential data designed to solve the problem of gradient vanishing in traditional Recurrent Neural Network. Compared with traditional Recurrent Neural Network, GRU has better long-term dependency modeling capability and higher computational efficiency, and its main feature is the introduction of two gating units, reset gate and update gate, which decide how the information flows through the sequence by learning. The reset gate controls the effect of the previous moment's hidden state on the current moment's inputs. In contrast, the update gate determines how much information is retained by the hidden state of the previous moment to be passed on to the next moment. The structure of GRU is shown in Fig. 1a. The data transfer process of GRU can be described as follows:

6 $$ {z}_{{t}} { = \sigma }\left( {{U}_{{z}} {f}_{{t}} { + w}_{{z}} {h}_{t - 1} { + b}_{{z}} } \right) $$

7 $$ {r}_{{t}} { = \sigma }\left( {{U}_{{r}} {f}_{{t}} { + W}_{{r}} {h}_{t - 1} { + b}_{{r}} } \right) $$

8 $$ {c}_{{t}} { = tanh}\left( {{U}_{{c}} {f}_{{t}} { + r}_{{t}} \left( {{W}_{{c}} \odot {h}_{t - 1} } \right){ + b}_{{c}} } \right) $$

9 $$ {h}_{{t}} { = }\left( {1 - {z}_{{t}} } \right) \odot {c}_{{t}} { + z}_{{t}} \odot {h}_{t - 1} $$

$\sigma$ denotes the ${sigmoid}$ activation function, ${tanh}$ denotes the hyperbolic tangent function, ${f}_{{t}}$ is the input vector per unit time, ${h}_{{t}}$ and ${h}_{t - 1}$ are the outputs of times $t - 1$ and ${t}$, respectively. ${z}_{{t}}$ and ${r}_{{t}}$ are the outputs of the update gate and reset gate, respectively, as in Eqs. 6 and 7 above, and ${c}_{{t}}$ is the candidate state, as in Eq. 8 above.${U}_{{z}} {,U}_{{r}}$ and ${U}_{{c}}$ are the connectivity matrices of the update gate, reset gate, and candidate states to the inputs, respectively.${W}_{{z}} {,b}_{{z}} {,W}_{{r}} {,b}_{{r}} {,W}_{{c}} {,b}_{{c}}$ are the weights and deviations of the update gate, reset gate, and candidate state, respectively. $\odot$ for the dot product operation.

The GRU transmission direction is unidirectional from front to back. However, the temporal data correlation is strong; the current moment state is related to the previous moment state and the next moment state. Therefore, for the problem of air pollutant concentration prediction, it is necessary to study the inverse time series and apply the BiGRU network to air pollutant concentration prediction. The BiGRU function combines the hidden layer states by developing two different loop layers, forward and backward, and the base structure of BiGRU is shown in Fig. 1b. Assuming that the input time series has a time window of size ${d}$, The input to the forward GRU is ${f}_{{t}} \left( {{t = 1,2,}...{,d}} \right)$ after the forward iteration, The forward output sequence of the implicit layer is shown in Eq. (10).

10 $$ \overrightarrow {{{h}_{{t}} }} { = }\overrightarrow {{{GRU}}} \left( {\overrightarrow {{{h}_{{{t - 1}}} }} {,f}_{{t}} } \right)\left( {{t = 1,2,}...{,d}} \right) $$

$\overrightarrow {{{GRU}}}$ denotes the forward mapping relation of the GRU. The input sequence ${f}_{{t}} \left( {{t = d,d - 1,}...{,1}} \right)$ reverses input for the reversed GRU is shown in Eq. (11).

11 $$ \overleftarrow {{{h}_{{t}} }} { = }\overleftarrow {{{GRU}}} \left( {\overleftarrow {{{h}_{{{t + 1}}} }} {,f}_{{t}} } \right)\left( {t = d,d - 1, \ldots ,1} \right) $$

where, $\overleftarrow {{{GRU}}}$ is the mapping relation of the backward GRU. Combining the above equations, the output ${h}_{{t}}$ of the hidden layer when ${t}$ is shown in Eq. (12).

12 $$ {h}_{{t}} { = }\left[ {\overrightarrow {{{h}_{{t}} }} {,}\overleftarrow {{{h}_{{t}} }} } \right] $$

**Figure 4:**
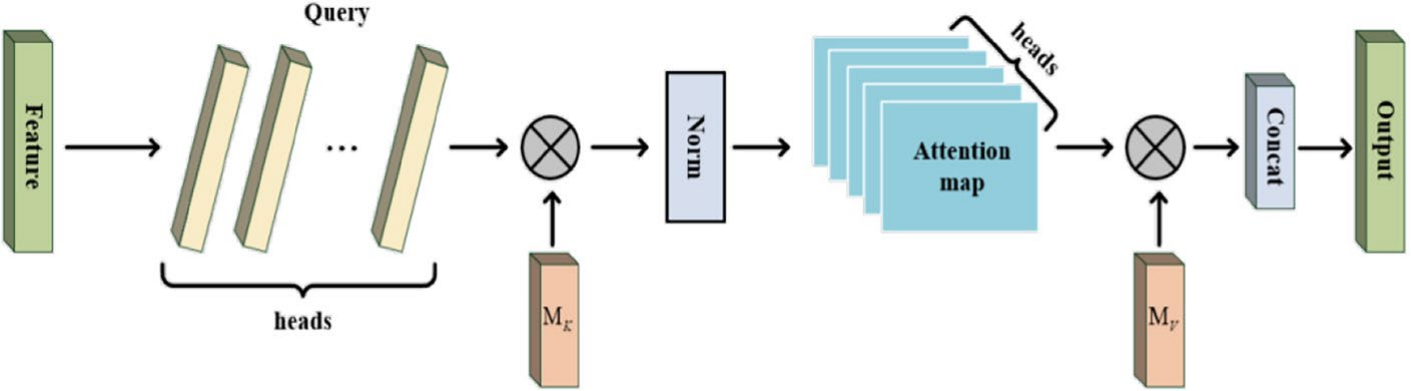
Multiple external attention mechanism structure.

In order to adaptively match the distribution between BiGRU units in each period while capturing the temporal dependency, a multi-head external attention mechanism is introduced to allocate enough attention to the critical information output from the implicit layer of the BiGRU network to learn the essential local information, as shown in Fig. 4 below. The output of the BiGRU layer is characterized by a matrix of ${F} \in {R}^{{{N} \times {d}}}$, where ${N}$ is the number of features affecting the parameter and ${d}$ is the dimension of the feature. The self-attention mechanism linearly maps this input to a query matrix ${Q} \in {R}^{{{m} \times {d}_{{k}} }}$, key matrix ${K} \in {R}^{{{m} \times {d}_{{k}} }}$, and the value matrix ${V} \in {R}^{{{m} \times {d}_{v} }}$. However, in practical applications, we often use two different memory cells ${M}_{{K}}$ and ${M}_{{v}}$ as keys and values in order to increase the size of the network capacity, and the single-head external attention matrix is shown in Eq. (13).

13 $$ \begin{aligned} {A} & { = }\left( {a} \right)_{{{i,j}}} { = Norm}\left( {{FM}_{{K}}^{{T}} } \right) \\ {F}_{{{out}}} & { = AM}_{{v}} \\ \end{aligned} $$

where ${M}_{k}$ and ${M}_{v}$ are learnable parameters, functioning as a memory. The external attention $\left( {a} \right)_{{{i,j}}}$ is the similarity between the ${i}$ feature and the ${j}$ row of the ${M}$. Update the input features of the external storage unit based on the similarity of the attention matrix. Based on the above single-head external attention mechanism, the multi-head external attention mechanism can be obtained by computing the attention multiple times on the outputs of different BiGRU units. The ith external attention is shown in Eqs. (14) and (15).

14 $$ {h}_{{i}} { = External}\,{Attention}\left( {{F}_{{i}} {,M}_{{k}} {,M}_{{v}} } \right) $$

15 $$ {F}_{{{out}}} { = MultiHead}\left( {{F,M}_{{K}} {,M}_{{v}} } \right){ = Concat}\left( {{h}_{{1}} {,}...{,h}_{{H}} } \right){W}_{{0}} $$

where ${h}_{{i}}$ is the $ith$ head, ${H}$ denotes the number of heads, ${W}$ is a linear transformation matrix, it is designed to keep the input and output dimensions consistent. ${M}_{{K}} \in {R}^{{{S} \times {d}}}$ and ${M}_{{\text{v}}} \in {R}^{{{S} \times {d}}}$ are used to compute the shared units of attention for each head.

### Transfer learning

Transfer learning^39^ is a method of learning by applying knowledge or models learned from one task to solve another related task. The domain, task, and marginal probabilities are used in transfer learning to describe transfer learning; the domain ${D}$ contains two parts, the feature space ${X}$, and the marginal probability distribution ${P}\left( {X} \right)$, as shown in Eq. (16).

16 $$ {D = }\left\{ {{x,P}\left( {X} \right)} \right\} $$

On the other hand, task ${\text{T}}$ also contains two parts, the feature space $\upgamma$, and the objective function ${f}\left( \cdot \right)$, as shown in Eq. (17).

17 $$ {T = }\left\{ {{\gamma ,f}\left( \cdot \right)} \right\} $$

where ${f}\left( \cdot \right)$ is obtained by learning from the training sample $\left\{ {{x}_{{i}} {,y}_{{i}} } \right\}$.

**Figure 5:**
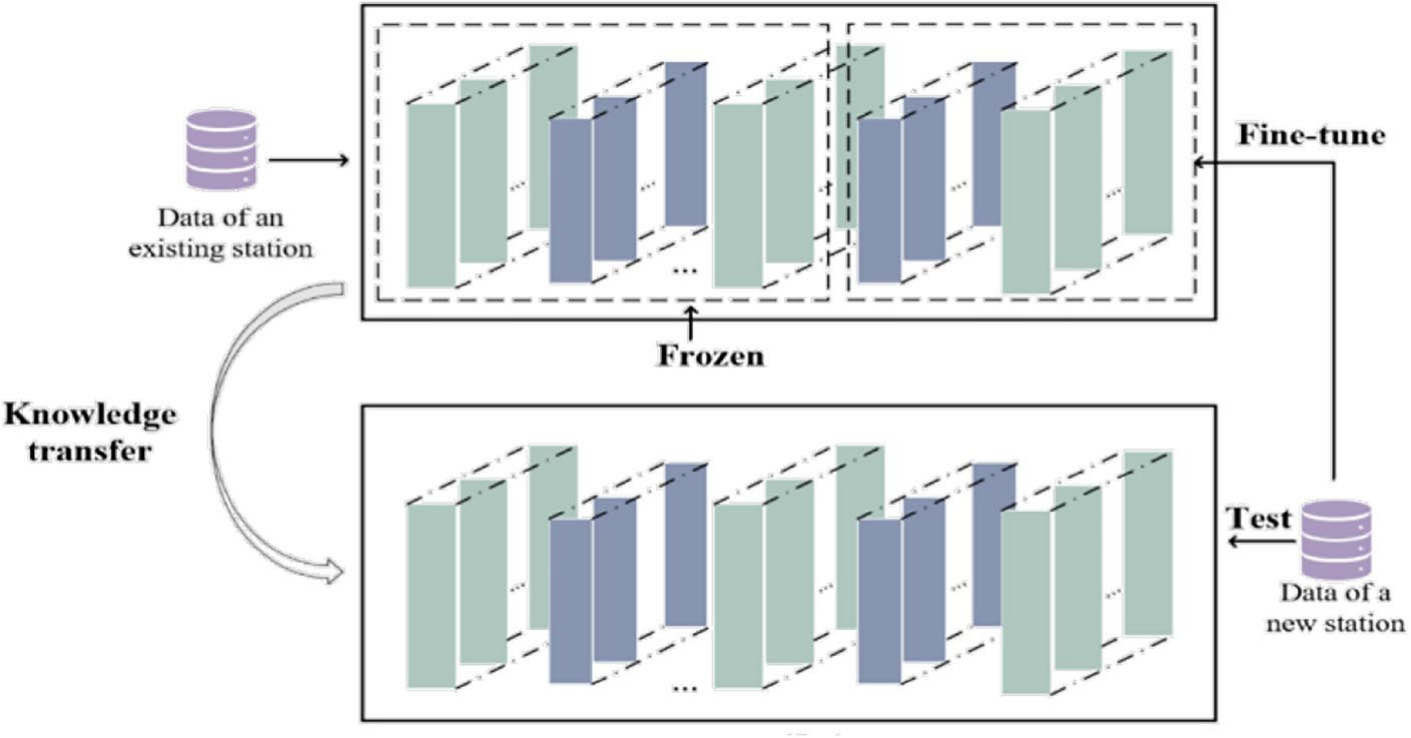
The working process of transfer learning.

The idea of transfer learning is to improve the prediction accuracy on the target domain task ${T}_{{T}}$ and target domain ${D}_{{T}}$ by utilizing the relevant knowledge learned from the source domain ${D}_{{S}}$ and the source task ${T}_{{S}}$, where ${\text{D}}_{S} \ne D_{T} ,T_{S} \ne T_{T}$. The schematic diagram is shown in Fig. 5 below.

The primary transfer learning methods can be divided into three categories: instance transfer learning, feature transfer learning, and model transfer learning. Instance transfer learning assigns high weights to samples with highly similar data distributions in the source and target domains, which accomplishes the transfer learning process. Feature information transfer learning is used to obtain the feature representation of inter-domain data in the relevant feature space so that the inter-domain data distribution differences are more similar than data feature extraction, and then the transfer learning process is completed. Model parameter transfer learning, on the other hand, is more intuitive and involves retaining the main structural hyper-parameters of the original model and then performing layer-specific fine-tuning of the parameters adapted to the target domain data, thus completing the transfer learning process.

This paper uses model parameter transfer learning, where knowledge in the source domain is shared with the target domain task for transfer. The specific process is as follows: firstly, freeze the last four layers of the model and train the network in the source domain data, and after training a certain amount of Epoch, observe the fitting effect of the model and retain the model parameter information; then, unfreeze the frozen layers to add a new fully-connected layer, and fine-tune the parameters of the fully-connected layer by using the data from the target domain to get the final atmospheric pollutant prediction model for the target site.

### Description of the algorithm

In order to facilitate the design and implementation of the proposed air pollutants prediction approach, the necessary steps are summarized as Algorithm 1 in this paper.

**Algorithm 1:**
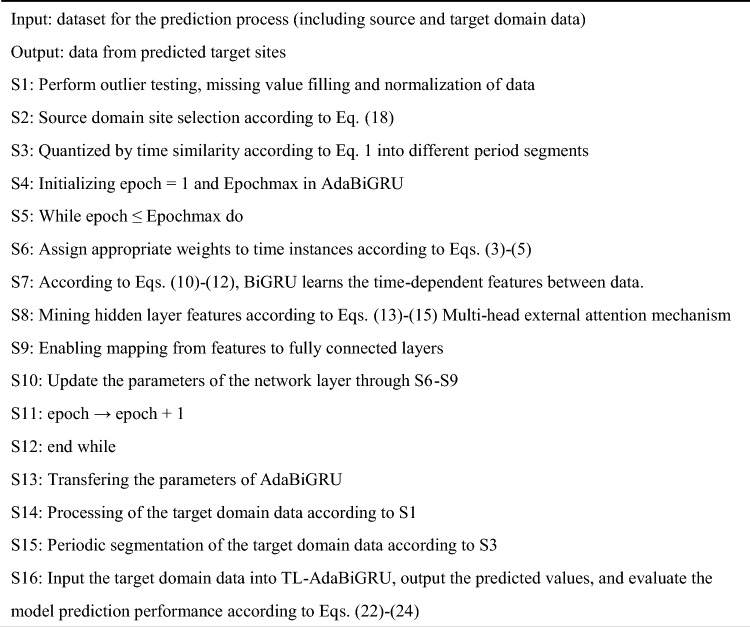
Air pollutants prediction via TL-AdaBiGRU

## Case study

### Dataset description and preprocessing

**Figure 6:**
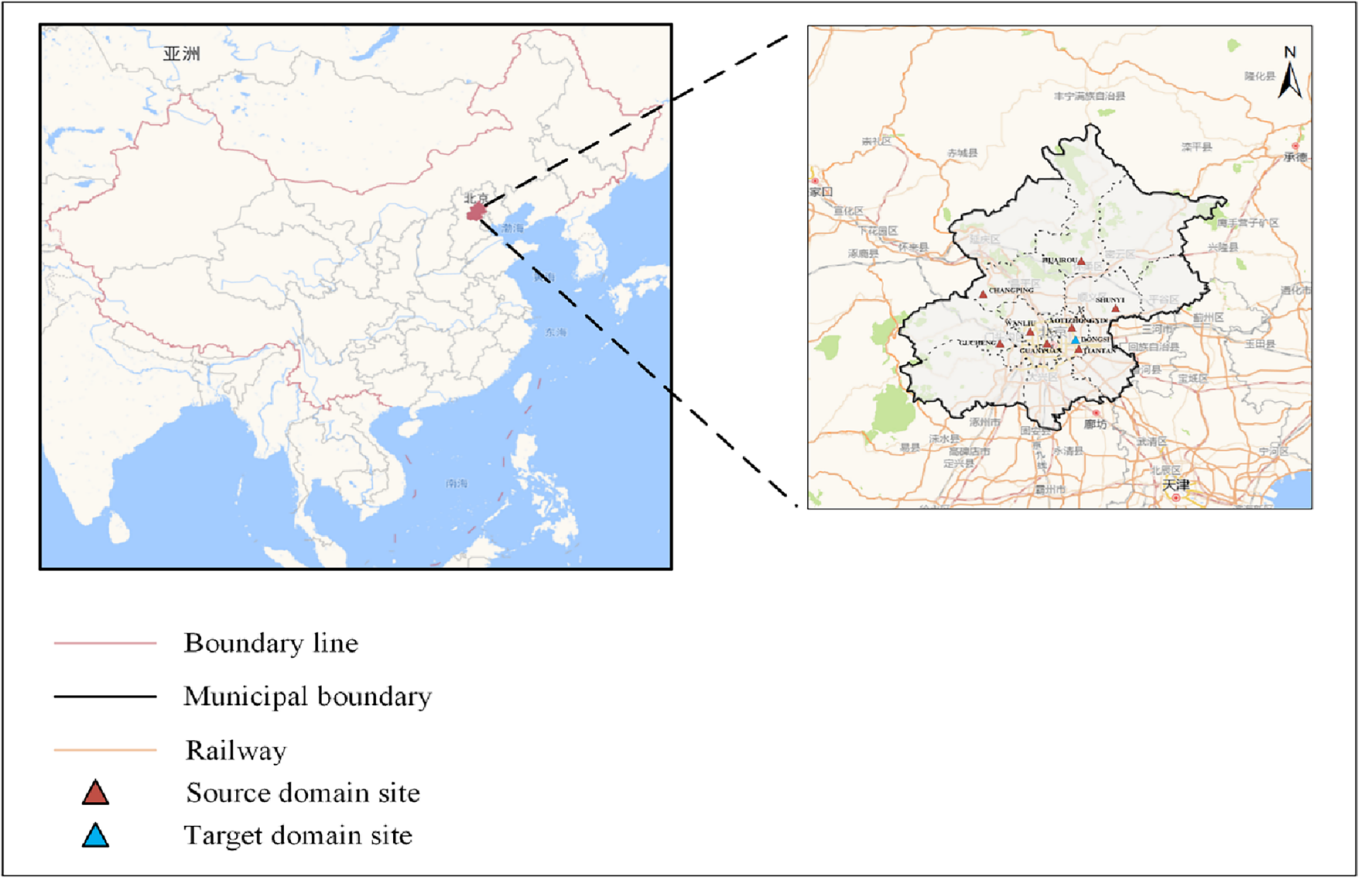
Distribution of the locations of the nine atmospheric monitoring stations in Beijing. Blue triangles represent stations with less historical data, and red triangles represent source domain stations with sufficient data. (This Figure is drawn by using Microsoft Visio software, the version number is 16.0.10730.20102 and the link to the software is http://officecdn.microsoft.com/pr/492350f6-3a01-4f97-b9c0-c7c6ddf67d60/media/zh-cn/VisioPro2019Retail.img).

Over the past few decades, Beijing has experienced rapid urbanization, industrial production, and energy consumption; however, this growth has also resulted in severe air pollution problems. A large number of pollutants are emitted every year, leading to a continuous decline in atmospheric quality. In this paper, the Beijing Municipality in China was selected as the study area, and the dataset was obtained from the Beijing Embassy in Foreign Countries (http://archive.ics. uci.edu/ml/datasets/Beijing+Multi-Site+Air-Quality+Data) 9 sites from March 2013 to February 2017 for atmospheric quality information. The locations of the atmospheric monitoring stations in this paper are shown in Fig. 6 below.

**Figure 7:**
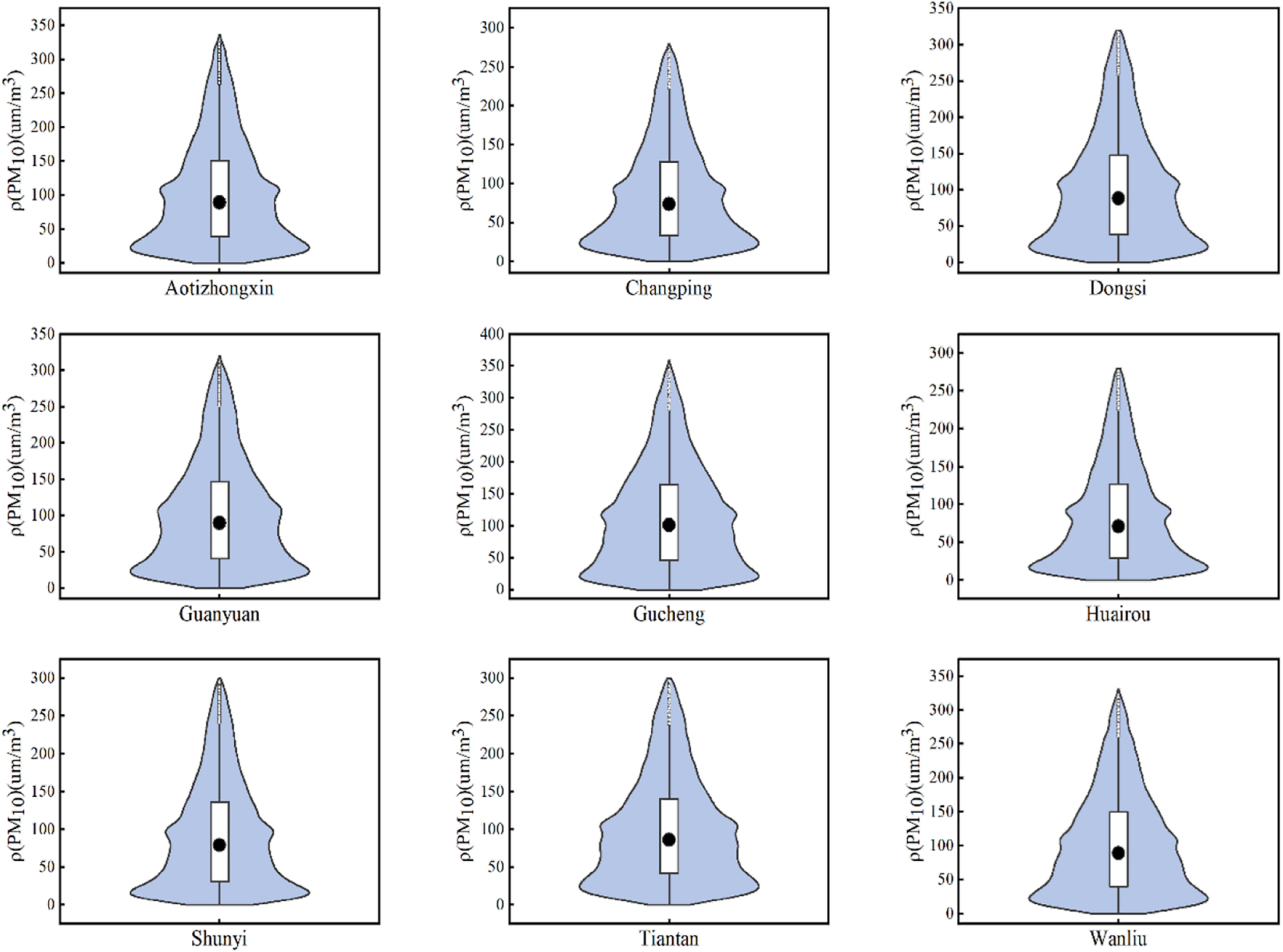
A violin plot with box plots showing the distribution of PM_10_ data at each site, with a maximum value set and data exceeding the maximum value identified as outliers.

**Figure 8:**
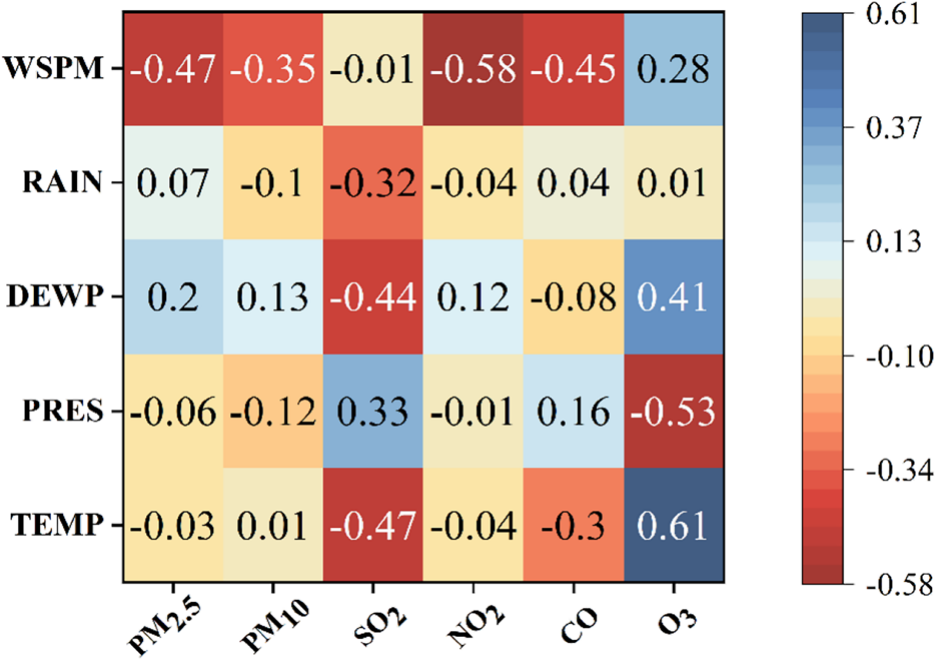
Spearman's correlation coefficient between pollutants and meteorological data. The Spearman correlation coefficient values range from − 1 to 1. The larger the absolute value of the coefficient, the stronger the correlation between the two variables.

In this study, PM_10_ was selected as the prediction target, and in order to characterize the distribution of PM_10_, a violin plot with a box shape was created with PM_10_ at each station, as shown in Fig. 7 below. The distribution of PM_10_ data at each site can be observed in the figure, and the maximum value is set in the violin plot; in this paper, the data more significant than the maximum value is called anomalous data, and the anomalous data is recorded as missing values. For PM_10_ concentration series data, the inconsistency of time stamps affects the prediction accuracy. Therefore, a linear interpolation algorithm is used to fill in the missing data, and the linear interpolation processed data is closer to the original data than the average interpolation method. In order to eliminate the dimensionality effect of the features and to improve the efficiency of the model operation, the maximum-minimum normalization method is used to make the data mapped in the same range. Atmospheric pollutants not only affect each other, but temperature and barometric pressure also have a strong influence on the pollutant effects; we plotted the Spearman correlation coefficient heat map as shown in Fig. 8, in which the temperature is negatively correlated with PM_2.5_, SO_2_, CO, and positively correlated with PM_10_, CO, PM_2. 5_, SO_2_, and NO_2_ are positively correlated with the barometric pressure. The dew-point temperature is correlated with PM_2.5_, PM_10_, NO_2_, and O_3_ were positively correlated, and negatively correlated with SO_2_ and CO. Rainfall showed a positive correlation with PM_2.5_, CO and O_3_, negative correlation with SO_2_ and NO_2_, wind speed was positively correlated with O_3_ and negatively correlated with the remaining five pollutants. The overall correlation between atmospheric pollutants and meteorological factors in the thermograms is weak, so the meteorological factors are entered as input layers with the auxiliary of the model input parameters.

### Source domain site selection

The purpose of this study is to explore the impact of transfer learning on the predictive performance of sites lacking historical data, the paper selected the Dongsi monitoring site as the target site, and the 6-month data from 2016/1 to 2016/7 was selected as the Dongsi site dataset. The limited historical data at the Dongsi site does not satisfy the need for deep learning model convergence. Therefore, in addition to the general features in the transfer pre-training model, source domain data are still needed to assist in learning the features of the target task, and the source domain monitoring sites play a crucial role in transferring the meteorological and temporal knowledge to the target domain sites. In this paper, we adopt the Maximum Mean Discrepancy (MMD) method to measure the similarity between the source domain monitoring sites and the target monitoring sites. The MMD method can efficiently measure the scatter of first-order distributions in the Reproducing Kernel Hilbert Space (RKHS). Datasets ${A = }\left\{ {{a}_{{i}} } \right\}_{{{i = 1}}}^{{{n}_{{1}} }}$ and ${B = }\left\{ {{b}_{{i}} } \right\}_{{{i = 1}}}^{{{n}_{{2}} }}$. The MMD of ${A}$ and ${B}$ is shown in Eq. (18).

18 $$ {MMD}_{{H}} \left( {{A,B}} \right){ = sup}\left( {{E}_{{p}} \left[ {{\Phi }\left( {a} \right)} \right] - {E}_{{q}} \left[ {{\Phi }\left( {b} \right)} \right]} \right) $$

where ${H}$ denotes the RKHS,${\Phi }\left( \cdot \right)$ is the nonlinear mapping function from the original data space to the RKHS, and ${p}$ and ${q}$ denote the probability distributions of the two datasets. MMD is further squared to obtain more precise results as shown in Eq. (19).

19 $$ \begin{aligned} {MMD}_{{H}}^{{2}} \left( {{A,B}} \right) & { = }\left\| {\frac{{1}}{{{n}_{{1}} }}\sum\nolimits_{{{i = 1}}}^{{{n}_{{1}} }} {{\Phi }\left( {{a}_{{i}} } \right) - \frac{{1}}{{{n}_{{2}} }}\sum\nolimits_{{{j = 1}}}^{{{n}_{{2}} }} {{\Phi }\left( {{b}_{{i}} } \right)} } } \right\|_{{H}}^{{2}} \\ & = \frac{{1}}{{{n}_{{1}}^{{2}} }}\sum\nolimits_{{{i = 1}}}^{{{n}_{{1}} }} {\sum\nolimits_{{{j = 1}}}^{{{n}_{{1}} }} {{k}\left( {{a}_{{i}} {,a}_{{j}} } \right)} } - \frac{{2}}{{{n}_{{1}} {n}_{{2}} }}\sum\nolimits_{{{i = 1}}}^{{{n}_{{1}} }} {\sum\nolimits_{{{j = 1}}}^{{{n2}}} {{k}\left( {{a}_{{i}} {,b}_{{j}} } \right)} } { + }\frac{{1}}{{{n}_{{2}}^{{2}} }}\sum\nolimits_{{{i = 1}}}^{{{n}_{{2}} }} {\sum\nolimits_{{{j = 1}}}^{{{n}_{{2}} }} {{k}\left( {{b}_{{i}} {,b}_{{j}} } \right)} } \\ \end{aligned} $$

The Gaussian Radial Basis Function (RBF) ${k}\left\langle {{a}_{{i}} {,b}_{{j}} } \right\rangle { = exp}\left( { - \left\| {{a}_{{i}} - {b}_{{j}} } \right\|^{{2}} {/2\gamma }^{{2}} } \right)$ is used where $k\left\langle { \cdot , \cdot } \right\rangle$ is the kernel function. Many studies have shown that multi-core MMD methods can improve domain adaptation^40^, and the kernel representation of ${{\text{N}}}_{{\text{k}}}{\text{RBF}}$ is as follows.

20 $$ {K}\left( {{a}_{{i}} {,b}_{{j}} } \right){ = }\sum\nolimits_{{{i = 1}}}^{{{N}_{{K}} }} {{k}_{{i}} \left( {{a}_{{i}} {,b}_{{j}} } \right)} $$

where ${k}_{{i}}$ denotes the RBF kernel with bandwidth parameter ${\gamma }_{{i}}^{{2}}$, the MMD between the source domain site and the target site is shown in Eq. (21).

21 $$ \begin{aligned} {MMD}_{{H}}^{{2}} \left( {{A,B}} \right) & { = }\left\| {\frac{{1}}{{M}}\sum\nolimits_{{{i = 1}}}^{{M}} {{\Phi }\left( {{A}_{{i}}^{{S}} } \right)} - \frac{{1}}{{{n}_{{{t,lab}}} }}\sum\nolimits_{{{j = 1}}}^{{{n}_{{t}} {,lab}}} {{\Phi }\left( {{A}_{{j}}^{{t}} } \right)} } \right\|_{{H}}^{{2}} \\ & = \frac{{1}}{{{M}^{{2}} }}\sum\nolimits_{{{i = 1}}}^{{M}} {\sum\nolimits_{{{j = 1}}}^{{M}} {{K}\left( {{A}_{{i}}^{{S}} {,A}_{{j}}^{{S}} } \right)} } - \frac{{2}}{{{Mn}_{{{t,lab}}} }}\sum\nolimits_{{{i = 1}}}^{{M}} {\sum\nolimits_{{{j = 1}}}^{{{n}_{{{t,lab}}} }} {{K}\left( {{A}_{{i}}^{{S}} {,A}_{{j}}^{{t}} } \right)} { + }\frac{{1}}{{{n}_{{{t,lab}}}^{{2}} }}\sum\nolimits_{{{i = 1}}}^{{{n}_{{{t,lab}}} }} {\sum\nolimits_{{{j = 1}}}^{{{n}_{{{t,lab}}} }} {{K}\left( {{A}_{{i}}^{{t}} {,A}_{{j}}^{{t}} } \right)} } } \\ \end{aligned} $$

where ${M}$ is the total number of source domain site samples.

The smaller the value of MMD, the higher the similarity with the target site; the results are shown in Table 1. The MMD values of Tiantan, Shunyi, Changping, and Dongsi are 0.669, 0.668, 0.667 respectively, and the MMD values of Guanyuan, Huairou, and Wanliu are 0.674, 0.657, 0.656 respectively, the above MMD values are all bigger than that of the Aotizhongxin value. Therefore, we selected the Aotizhongxin site as the source domain data set. The site, auxiliary target site, and the data of the Aotizhongxin site for 42 months from 2013/1 to 2016/7 were selected as the source domain dataset. The descriptive data statistics of the Dongsi site (target site) and the Aotizhongxin site (source domain site) are shown in the following Table 2.

**Table 1:** MMD values between target atmospheric monitoring sites and neighboring atmospheric monitoring sites.

Station	Aotizhongxin	Changping	Guanyuan	Gucheng	Huairou	Shunyi	Tiantan	Wanliu
Dongsi	0.647	0.667	0.674	0.663	0.657	0.668	0.669	0.656

**Table 2:** The descriptive data statistics of the target site and the source domain site.

Station	Record count	Variables	Mean	Standard deviation	Minimum	Maximum
Dongsi (target domain)	5112	PM_10_	89.328	64.450	5.000	318.000
		PM_2.5_	67.245	56.502	3.000	258.000
		SO_2_	11.858	11.917	2.000	54.000
		NO_2_	43.614	26.770	6.000	138.000
		CO	977.928	648.754	100.000	3100.000
		O_3_	69.345	54.174	2.000	304.000
		TEMP	13.995	12.415	16.800	37.300
		PRES	1011.391	11.058	990.100	1042.000
		DEWP	− 0.016	15.011	− 35.300	27.300
		RAIN	0.086	0.977	0.000	24.100
		WSPM	2.098	1.235	0.000	8.100
Aotizhongxin (Source domain)	29,976	PM_10_	108.610	93.880	2.000	491.000
		PM_2.5_	84.493	83.321	3.000	537.000
		SO_2_	19.463	23.782	0.286	278.000
		NO_2_	52.484	33.291	2.000	258.000
		CO	1292.583	1120.248	100.000	6000.000
		O_3_	60.373	59.411	0.643	671.000
		TEMP	14.280	11.357	− 16.800	41.100
		PRES	1011.751	10.189	987.100	1042.000
		DEWP	2.807	13.772	− 35.300	28.800
		RAIN	0.066	0.777	0.000	36.600
		WSPM	1.897	1.304	0.000	10.500

## Result

### Model parameters and evaluation indicators

According to the Table 1 results with the Aotizhongxin site as the source domain site, the data of 42 months from 2013/1 to 2016/7 are collected as the source domain dataset for model pre-training. 80% of its data are used as the training set, 10% as the testing set, and 10% as the validation set. The source domain site data are input into AdaBiGRU after outlier detection, missing value filling and normalization, period segmentation by the TDC layer, and allocation of different weights by temporal self-attention mechanism. In this paper, the lag time is set to 24 h, the Dropout is 0.5, and the model is optimized using Adam optimizer with a learning rate of 0.005, Batch size set to 36, activation function of Relu, and loss function of MSE. In this paper, we utilize the root mean squared error (RMSE), the mean absolute error (MAE), and the mean absolute percentage error (MAPE) as three evaluation metrics to evaluate the prediction performance of AdaBiGRU. The formulas for these three metrics are as follows.

22 $$ {RMSE = }\sqrt {\frac{{1}}{{n}}\sum\nolimits_{{{i = 1}}}^{{n}} {\left( {{y}_{{i}} - {y}_{{i}}^{{*}} } \right)}^{{2}} } $$

23 $$ {MAE = }\frac{{1}}{{n}}\sum\nolimits_{{{i = 1}}}^{{n}} {\left| {{y}_{{i}} - {y}_{{i}}^{{*}} } \right|} $$

24 $$ {MAPE = }\frac{{1}}{{n}}\sum\nolimits_{{{i = 1}}}^{{n}} {\frac{{\left| {{y}_{{i}} - {y}_{{i}}^{{*}} } \right|}}{{{y}_{{i}} }}} $$

where ${n}$ denotes the number of samples,${y}_{{i}}$ denotes the observed value of the *i*-th sample, and ${y}_{{i}}^{{*}}$ denotes the predicted value of the *i*-th sample. The smaller the value of these three indicators, the higher the prediction accuracy and the better the model's performance.

### Comparison of pre-trained models

In order to test the performance of the AdaBiGRU model, this paper compares it with five prediction models, namely, ARIMA, GRU, BiGRU, LightGBM, and Transformer, at four sites, namely, the Gucheng, the Tiantan, the Aotizhongxin, and Wanliu, and the results are shown in Table 3. For PM_10_ concentration, the error values of both ARIMA and LightGBM are higher than those of GRU, BiGRU, Transformer, and AdaBiGRU, which suggests that the time-series neural network model has higher prediction accuracy in atmospheric quality prediction. BiGRU predicts better than GRU. The performance of the Transformer is better than GRU and BiGRU, indicating that the model based on the attention mechanism performs better than the traditional model. In addition, the proposed AdaBiGRU model has smaller values than GRU, BiGRU, and Transformer, proving that AdaBiGRU is effective when applied to the problem of atmospheric pollutant concentration prediction.

**Table 3:** Comparison of effects of pre-trained models.

	Tiantan		Aotizhongxin		Wanliu		Gucheng	
	RMSE	MAE	RMSE	MAE	RMSE	MAE	RMSE	MAE
ARIMA	0.181	0.136	0.141	0.108	0.156	0.12	0.092	0.071
GRU	0.051	0.038	0.048	0.035	0.046	0.033	0.035	0.025
BiGRU	0.036	0.026	0.018	0.012	0.026	0.019	0.028	0.015
LightGBM	0.058	0.039	0.041	0.028	0.042	0.031	0.032	0.021
Transformer	0.034	0.022	0.033	0.016	0.027	0.018	0.026	0.016
AdaBiGRU	**0.030**	**0.019**	**0.021**	**0.014**	**0.022**	**0.014**	**0.025**	**0.015**

Significant values are in bold.

### TL-AdaBiGRU

**Figure 9:**
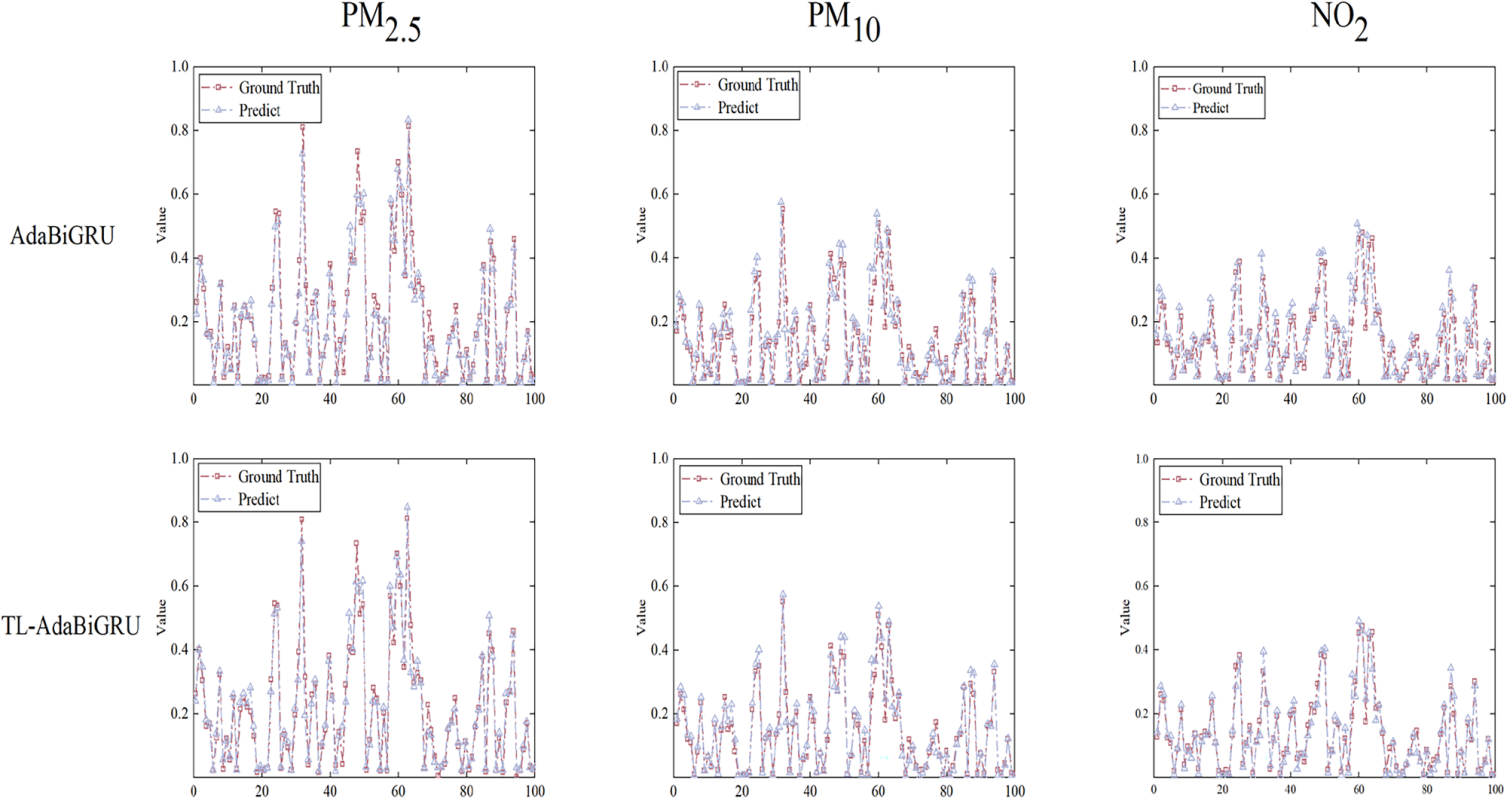
Comparison of actual and predicted values on PM_2.5_, PM_10_, and NO_2_ by AdaBiGRU and TL-AdaBiGRU models.

In order to improve the prediction performance of the model in limited data sites, this paper implements TL-AdaBiGRU by combining AdaBiGRU with model parameter transfer learning. The model is first trained on sufficient source domain datasets to determine the optimal model parameters; then, the last four layers of the model are frozen, and the model parameter information is retained after a certain amount of Epoch training. Finally, the frozen layers were unfrozen, and a new fully connected layer was added to fine-tune the source domain model using the target domain data to improve the prediction accuracy at the target site. The frozen layers of the model need to be identified before fine-tuning the model, which serves to preserve the knowledge learned by the pre-trained model on the source domain data and to prevent performance degradation due to over-tuning on the target domain data. The number of freezing layers directly affects the prediction performance of the model. If the number of freezing layers is too small, the model may not be able to learn enough “knowledge” from the source data. If the number of freezing layers is too large, the model will not be able to adjust enough parameters for the target data, which will affect the prediction effect. Therefore, to make the model have better prediction performance, selecting the appropriate number of freezing layers is a key issue. The AdaBiGRU model was pre-trained using PM_10_ concentration data from the Aotizhongxin site. Eighty percent of the samples collected from the Dongsi site for six months of data from 2016/1 to 2016/7 were used to fine-tune the model with different numbers of freezing layers; 10 percent was used for testing and 10 percent for validation. The results presented in Table 4 below show that the values of the three metrics decrease as the number of freezing layers increases, reaching a minimum when the number of freezing layers is 4. This is because when the number of freezing layers is too small, the model is affected by noise from other sites. As the number of frozen layers increases, the model is gradually less affected by noise from other sites, and the performance improves. When the number of frozen layers is more than 4, the error increases as the number of frozen layers increases, and this result is due to the overfitting of the model to the auxiliary sites. Therefore, this paper sets the number of frozen layers to 4. In order to verify the validity and reasonableness of the number of freezing layers of the model, we used the same method to experiment with the number of freezing layers of PM_2.5_ and NO_2_ pollutants and determined the optimal number of freezing layers is also four layers. After that, the transfer model was tested using 20% of the data from the Dongsi site, and the comparison between the predicted and real values is shown in Fig. 9. Compared with the AdaBiGRU model, the fitting effect of the TL-AdaBiGRU model is significantly improved.

**Table 4:** Impact of the number of frozen layers on prediction accuracy of model.

Frozen layers	RMSE	MAE	MAPE
1	0.1	0.072	0.103
2	0.084	0.065	0.076
3	0.048	0.034	0.055
4	0.029	0.021	0.035
5	0.054	0.043	0.057
6	0.081	0.065	0.073
7	0.11	0.083	0.092

## Discussions

The performance of the proposed methodological framework for atmospheric site prediction is presented in the previous sections. Its reliability and applicability still need to be further explored. This section focuses on the period segmentation of the time-similarity quantization algorithm, the validation of the model's effectiveness at other monitoring stations, and the prediction effectiveness of the proposed model for other pollutants.

### Time similarity quantization period segmentation

**Figure 10:**
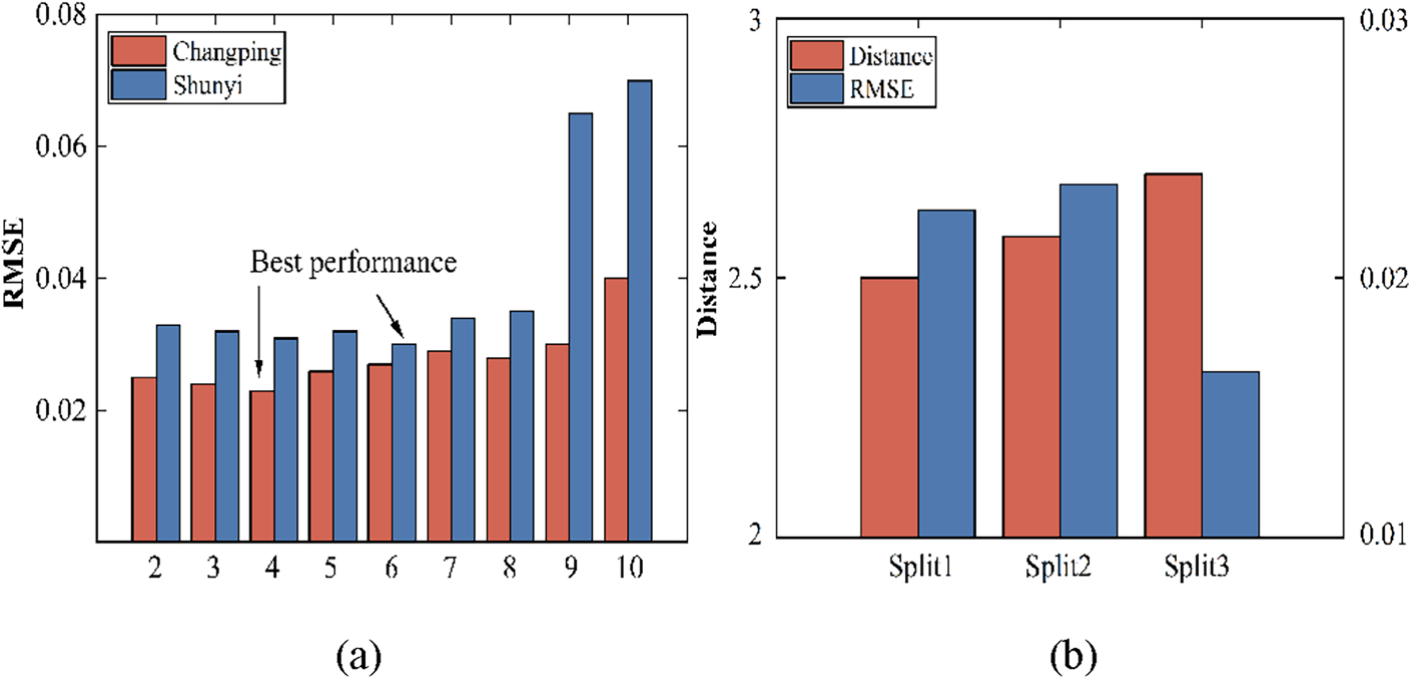
Comparison of quantitative results for temporal similarity.

In section temporal distribution characterization above for the air pollutant data is periodic and non-stationary, the data distribution changes dynamically over time; in order to better characterize the distribution information in the air pollutant series, this paper adopts dynamic programming (DP) to solve the optimization problem of Eq. (1). First, the time series is uniformly partitioned into ${N = 10}$ parts, each of which is the most minor unit period that cannot be subdivided. Then, the value of a is chosen randomly for ${\text{K}}$ range of values of ${K = }\left\{ {{2,3,4,5,6,7,8,9,10}} \right\}$. For a given value of ${\text{K}}$, a greedy strategy is used to choose the length ${n}_{{j}}$ of each period. Use ${\text{A}}$ and ${\text{B}}$ to denote the start and end points of the time series, respectively. First, consider the case of ${K = 2}$ and maximize the distribution distance ${d}\left( {{S}_{{{AC}}} {,S}_{{{CB}}} } \right)$ by choosing a segmentation point (denoted as ${\text{C}}$), specifically, choosing one of the ${\text{N}}$ segments as ${\text{C}}$ such that ${d}\left( {{S}_{{{AC}}} {,S}_{{{CD}}} } \right){ + d}\left( {{S}_{{{DB}}} {,S}_{{B}} } \right)$ is maximized. In this way, the time series is divided into three parts: $\left[ {{A,C}} \right]{,}\left[ {{C,D}} \right]$ and $\left[ {{D,B}} \right]$. Similarly, ${K = 4,5,6,7,8,9,10}$, the same strategy is used to maximize the distribution distance. With the greedy strategy, the optimal splitting point can be selected so that the length of each period of the time series can be more evenly distributed, thus obtaining a better prediction model performance. In order to verify the effectiveness of the proposed method, experiments were carried out at two sites, Changping and Shunyi, as shown in Fig. 10a below; with the increase of ${\text{K}}$, the model performance first becomes better and then worse, and the model performance is the best when ${K = 4,6}$ and the model performance gradually decreases with the increase of ${\text{K}}$. The model performance of ${K = 4,6}$ is the best, and the model performance gradually decreases with the increase of the ${K}$ value. In order to verify the effectiveness of temporal distribution characterization for segmentation of atmospheric pollutant sequences, comparative experiments were carried out as shown in Fig. 10b below; Split1 represents random partitioning, Split2 represents partitioning based on closest similarity, and Split3 represents partitioning quantified by temporal similarity. Our TDC divides the atmospheric pollutant sequence into the time periods with the greatest distribution distance, which means that RMSE is the best when partitioning into the least similar time periods.

### Validation of other monitoring sites

**Figure 11:**
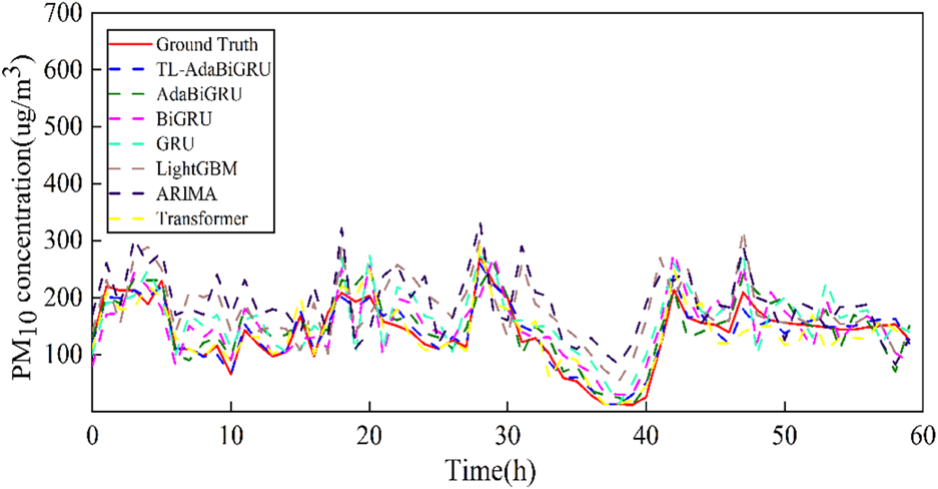
Comparison of different models at the Huairou monitoring site. The red solid line is the real value, the blue dotted line represents TL-AdaBiGRU, the green dotted line represents AdaBiGRU, the pink dotted line represents BiGRU, the indigo dotted line represents GRU, the brown dotted line represents LightGBM, the purple dotted line represents ARIMA, and the yellow dotted line represents Transformer.

**Figure 12:**
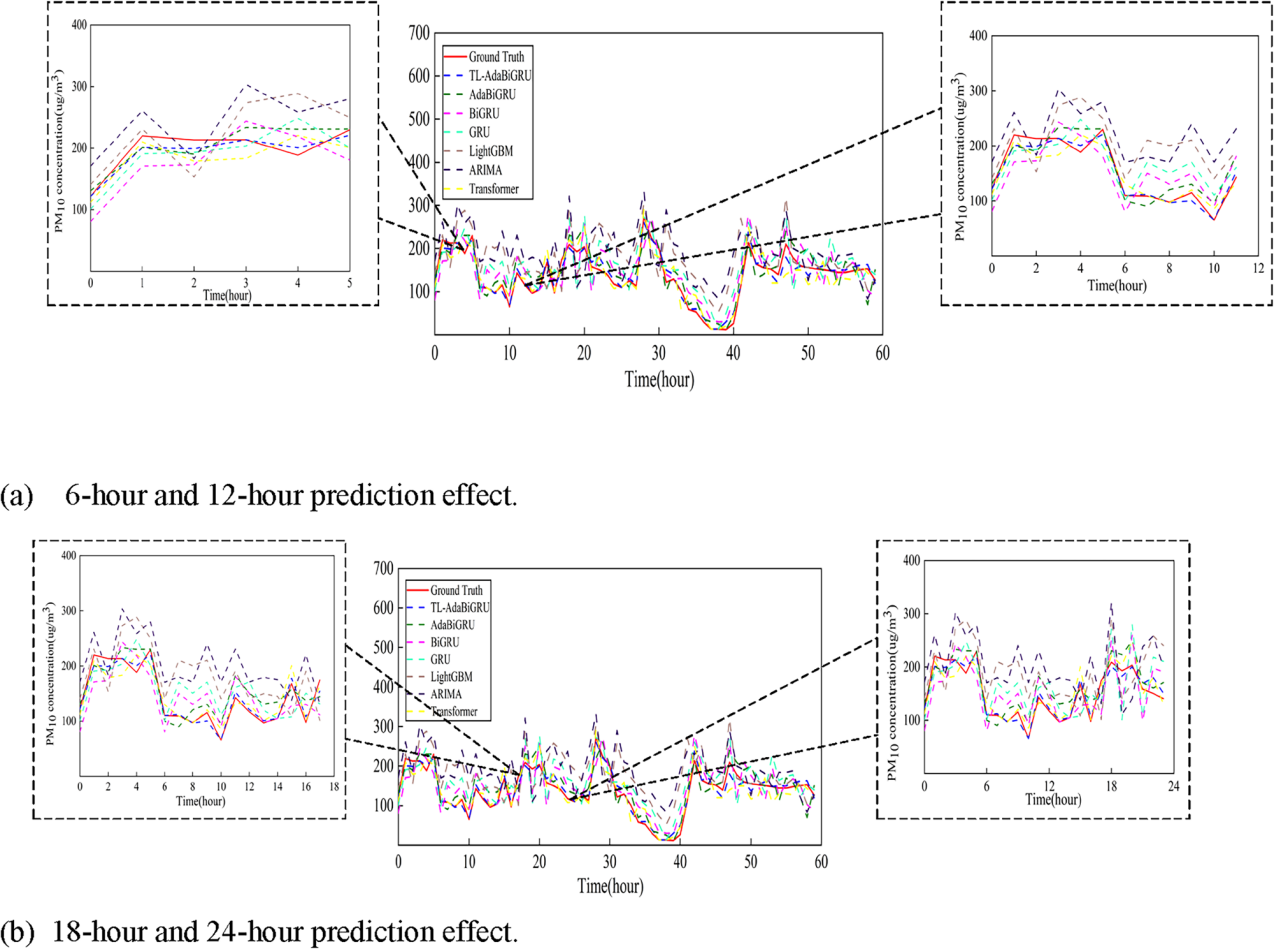
Comparison of the effects of multi-step prediction effects. (**a**) Figure shows the prediction effects of the models at 6 and 12 h. (**b**) Figure shows the prediction effects of the models at 18 and 24 h.

In order to verify the validity of the model proposed in this paper, we compared TL-AdaBiGRU with six models, namely, ARIMA, GRU, BiGRU, LightGBM, and Transformer, AdaBiGRU, at the Huairou monitoring site. We selected the 6-month data from 2016/6 to 2016/12 at the Huairou monitoring station as the dataset and predicted the PM_10_ concentration for 2017/1/1/0:00 a.m.–1/3/12:00 a.m. (60 h in total). It can be seen from Fig. 11 that with less data, the PM_10_ concentration predicted by the TL-AdaBiGRU model is closer to the actual value compared with the other models closer to the real value. The model effectively alleviates the problems of low prediction accuracy and weak generalization ability caused by the small amount of data. The model proposed in this paper is also very effective in multi-step prediction, predicting the next 6, 12, 18, and 24 h, as shown in Fig. 12.

### Predictive applications for other pollutants

**Figure 13:**
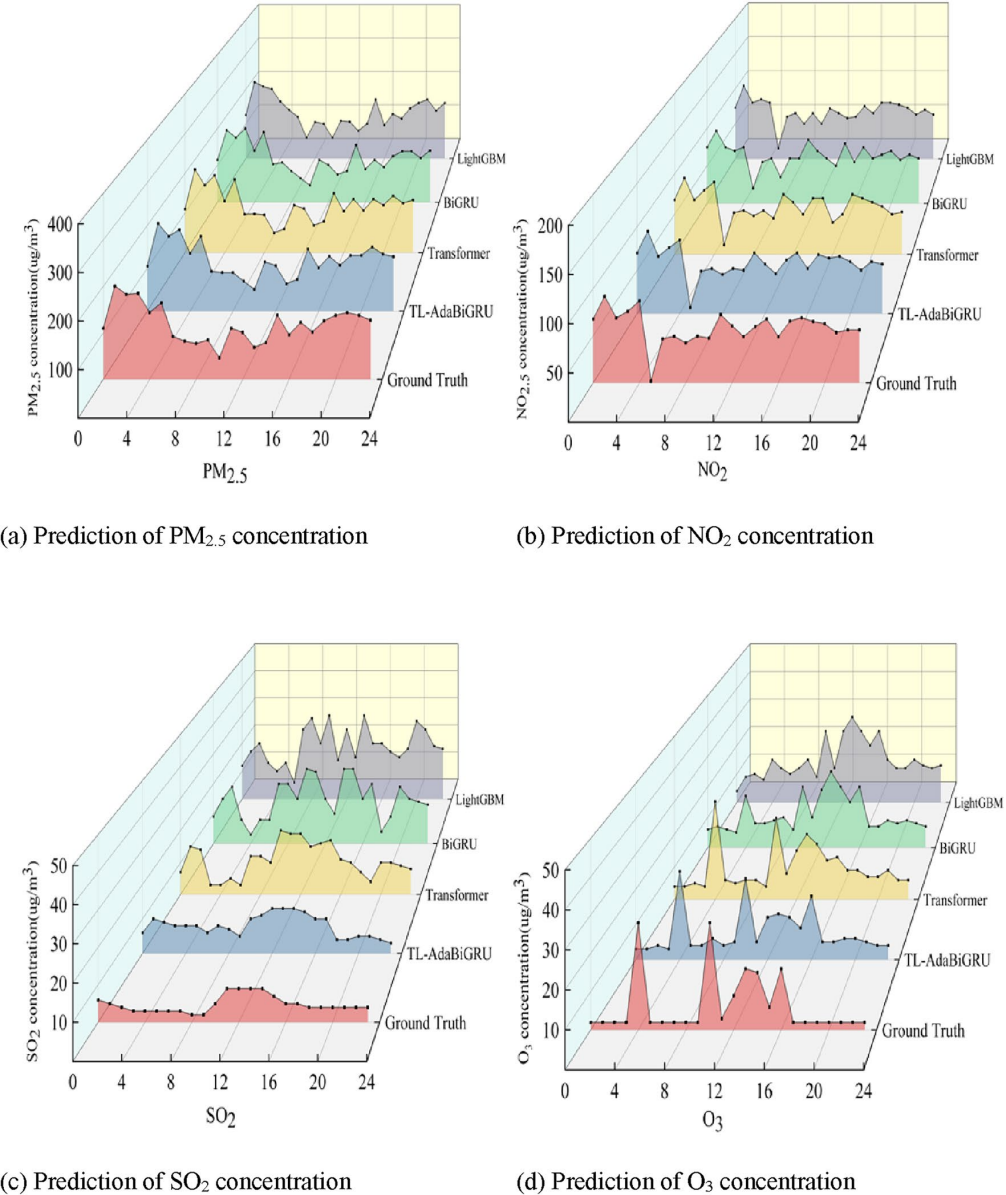
Predicted results of PM_2.5_, NO_2_, SO_2_ and O_3_ concentrations. The red part represents the real value, the blue represents the TL-AdaBiGRU model, the yellow represents the Transformer model, the green represents the BiGRU model, and the gray represents the LightGBM.

The TL-AdaBiGRU model proposed in this article has achieved high accuracy in predicting PM_10_ concentration. In order to further verify the generalization of the model, we used the dataset from Huairou Station to predict PM_2.5_, NO_2_, SO_2_, and O_3_ pollutants, as shown in Fig. 13. Our proposed model has shown good predictive performance on various pollutants, and experimental results have shown that the TL-AdaBiGRU model can effectively address the problem of low prediction accuracy caused by limited data volume.

## Conclusion

This paper proposes a two-stage attention mechanism model (TL-AdaBiGRU) based on transfer learning to improve the prediction accuracy of newly built monitoring sites with limited historical monitoring data. The model is first pre-trained using source domain sites with sufficient data. The data in the pre-training phase are processed by a temporal distribution characterization layer and then entered into a temporal distribution matching layer that integrates a temporal attention mechanism and a multi-head external attention mechanism. The temporal attention mechanism can adaptively select relevant sequences and assign weights, thus capturing the feature information of the input sequences. The multi-head external attention mechanism can dig deeper into the key features of the hidden layer of the network to quickly filter out the critical features among many inputs. After the two attention mechanisms, the model can not only adaptively select the most relevant input features but also efficiently capture the time dependence of the time series. Then, based on the pre-trained model, a fine-tuning strategy is used to freeze the last few layers of the pre-trained model and fine-tune the remaining layers using the target domain data. The fine-tuned model can transfer the knowledge learned at the source site to the target site, thus improving the prediction accuracy. In this paper, experiments were conducted using air pollutant data from Beijing, and the main results are as follows:

- Quantifying temporal distribution characterization can be an excellent way to deal with air pollutant concentration data characterized by periodicity and dynamic changes in data distribution over time.
- The two-stage attention mechanism of the model can better analyze the nonlinear relationship between the air pollutant data, and in the PM_10_ concentration prediction experiments, the prediction results of the TL-AdaBiGRU proposed in this paper are better than those of AdaBiGRU, Transformer, BiGRU, GRU and LightGBM.
- Transfer learning can effectively improve the performance of pollutant concentration prediction at data shortage sites, and other pollutant prediction experiments were conducted at data shortage sites with good results, verifying that the model has strong generalization.

The contribution of this study lies in the fact that a TL-AdaBiGRU model is proposed to solve the problem of the small amount of historical data of newly built air quality monitoring stations and the problem that the time series data of air pollutants have periodicity and the data distribution changes dynamically with time, and the prediction accuracy of the proposed model at newly built stations is significantly improved. Taking Beijing's air pollutant concentration data as an example, this paper proves that the model has higher accuracy. Of course, the method proposed in this paper also has limitations. Firstly, since the idea of transfer learning is to “learn from similar time series,” the current method can only rely on having similar sites to assist in learning the target. If there is no such a learning target, transferring learning is not feasible. Second, this study only predicted pollutant concentration data for a few cities, and the migration analysis of the model was not comprehensive enough. Future work could apply the model to predict pollutant concentrations in multiple areas. In addition, the model can be applied to studying other time-series data predictions, such as stock price predictions, power load data predictions, and traffic flow predictions. Third, although the method proposed in this paper improves the accuracy of pollutant prediction, its superior performance cannot be supported by high-quality data, especially under different geographic conditions and infrastructures, and its applicability needs to be further improved in future studies. In future studies, we will try to consider other aspects, such as combining the knowledge of meta-transfer learning, domain adaptation, and domain generalization, to consider the generalization and robustness of the model under different environments and infrastructures to further improve the overall performance of the prediction model.

## Data Availability

The datasets used and/or analysed during the current study available from the corresponding author on reasonable request.
